# Endoscopic retrograde direct cholangioscopy extracting stones from common bile duct in a case of ectopic duodenal papilla

**DOI:** 10.1055/a-2715-5246

**Published:** 2025-11-06

**Authors:** Shan-Shan Hu, Yun-Chao Yang, Jie Hou, Wei-Hui Liu

**Affiliations:** 1 89669 Department of Gastroenterology and Hepatology, Sichuan Provincial Peopleʼs Hospital, School of Medicine, University of Electronic Science and Technology of China, Chengdu, Sichuan Province, China

Ectopic opening of the duodenal papilla within the duodenal bulb is a rare anatomical anomaly, which may be associated with clinical conditions such as duodenal ulcer, duodenal stenosis, bile duct stones, or acute cholangitis 1 2 . Recently, we successfully utilized endoscopic ultrasonography combined with endoscopic retrograde direct cholangioscopy (ERDC) 3 4 to diagnose and treat a case of an ectopic duodenal papilla complicated by common bile duct stones.

A patient presenting with abdominal pain and common bile duct stones was evaluated. Gastroscopy revealed an ulcer in the duodenal bulb, accompanied by significant deformation and narrowing of the intestinal lumen. Endoscopic ultrasonography demonstrated an abnormally shortened distance of 12 mm between the pylorus and the papilla, as well as dilation and shortening of the common bile duct, distal ductal stenosis, and the presence of multiple intraductal stones ( Fig. 1 ). After the initial medical management of the ulcer, the patient underwent ERDC for stone removal. Duodenoscopy showed that the ulcer had healed with annular scar stenosis ( Fig. 2 ). The papillary orifice was not visible at its conventional anatomical position; however, guided by bile flow and longitudinal mucosal folds, an ectopic papilla was identified within the annular scar of the duodenal bulb. Due to the confined space in which traditional intubation methods may have difficulty precisely aligning with the bile duct opening, the ERDC direct visualization intubation technique can autonomously adjust its direction to achieve accurate insertion into the bile duct. ERDC was subsequently employed for direct visualization ( Fig. 3 ). Access to the common bile duct was achieved, balloon dilation was performed, and all stones were successfully extracted ( Fig. 4 , Video 1 ).

**Fig. 1:**
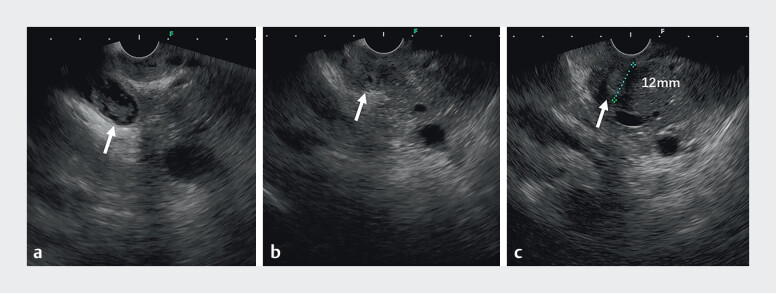
Endoscopic ultrasonography findings indicating an ectopic duodenal papilla. **a** The common bile duct is dilated and shortened, containing numerous stones. **b** Distal common bile duct stenosis. **c** The endoscopic ultrasound probe positioned at the pyloric orifice shows a pylorus-to-papilla distance of 12 mm.

**Fig. 2:**
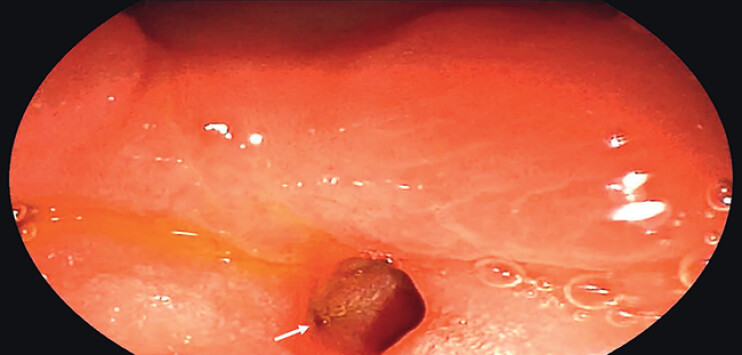
The orifice was identified ectopically within the duodenal bulb.

**Fig. 3:**
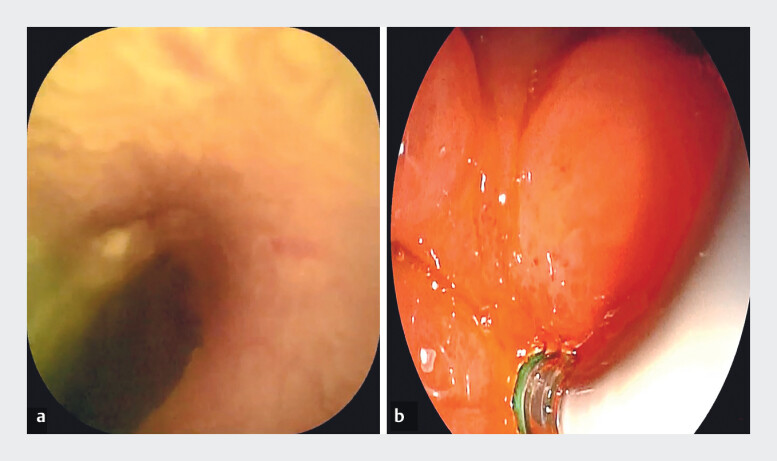
Achieving ectopic duodenal papilla cannulation with ERDC technology.

**Fig. 4:**
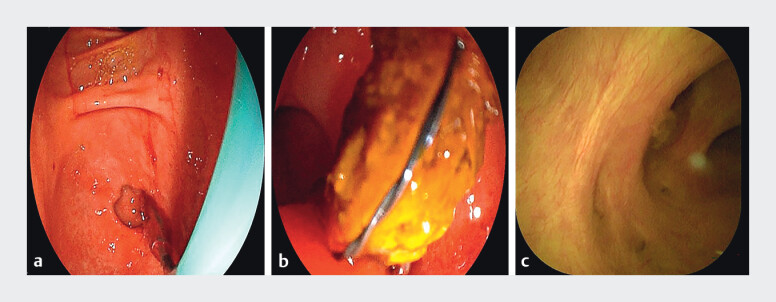
Stone extraction performed using ERDC technology. **a** Balloon dilation of the stenotic papillary orifice. **b** Direct visualization and stone extraction via choledochoscopy. **c** Postoperative evaluation confirming complete clearance of bile duct stones.

The first case of an ectopic duodenal papilla diagnosed and treated with full visual guidance through combined ERDC and EUS technology.Video 1

This case highlights the importance of considering the possibility of an ectopic papilla in patients with duodenal bulb ulcers and stenosis when the papilla is not located at its normal position. Endoscopic ultrasonography is valuable for identifying a shortened pylorus-to-papilla distance and characteristic changes in the common bile duct. At the same time, this marks the first case of an ectopic duodenal papilla diagnosed and treated with full visual guidance using ERDC technology.

Endoscopy_UCTN_Code_CCL_1AZ_2AK

## Notes

Endoscopy E-Videos https://eref.thieme.de/e-videos
*E-Videos* is an open access online section of the journal *Endoscopy* , reporting on interesting cases and new techniques in gastroenterological endoscopy. All papers include a high-quality video and are published with a Creative Commons CC-BY license. Endoscopy E-Videos qualify for HINARI discounts and waivers and eligibility is automatically checked during the submission process. We grant 100% waivers to articles whose corresponding authors are based in Group A countries and 50% waivers to those who are based in Group B countries as classified by Research4Life (see: https://www.research4life.org/access/eligibility/ ). This section has its own submission website at https://mc.manuscriptcentral.com/e-videos .
